# Cranberry extracts promote growth of *Bacteroidaceae* and decrease abundance of *Enterobacteriaceae* in a human gut simulator model

**DOI:** 10.1371/journal.pone.0224836

**Published:** 2019-11-12

**Authors:** Kathleen O’Connor, Madeleine Morrissette, Philip Strandwitz, Meghan Ghiglieri, Mariaelena Caboni, Haiyan Liu, Christina Khoo, Anthony D’Onofrio, Kim Lewis

**Affiliations:** 1 Antimicrobial Discovery Center, Department of Biology, Northeastern University, Boston, Massachusetts, United States of America; 2 Global Scientific Affairs and Nutrition Policy, Research and Development, Ocean Spray Cranberries, Inc., Lakeville-Middleboro, Massachusetts, United States of America; Universidade Catolica Portuguesa, PORTUGAL

## Abstract

The opportunistic pathogen *Escherichia coli*, a common member of the human gut microbiota belonging to the *Enterobacteriaceae* family, is the causative agent of the majority of urinary tract infections (UTIs). The gut microbiota serves as a reservoir for uropathogenic *E*. *coli* where they are shed in feces, colonize the periurethral area, and infect the urinary tract. Currently, front line treatment for UTIs consists of oral antibiotics, but the rise of antibiotic resistance is leading to higher rates of recurrence, and antibiotics cause collateral damage to other members of the gut microbiota. It is commonly believed that incorporation of the American cranberry, *Vaccinium macrocarpon*, into the diet is useful for reducing recurrence of UTIs. We hypothesized such a benefit might be explained by a prebiotic or antimicrobial effect on the gut microbiota. As such, we tested cranberry extracts and whole cranberry powder on a human gut microbiome-derived community in a gut simulator and found that cranberry components broadly modulate the microbiota by reducing the abundance of *Enterobacteriaceae* and increasing the abundance of *Bacteroidaceae*. To identify the specific compounds responsible for this, we tested a panel of compounds isolated from cranberries for activity against *E*. *coli*, and found that salicylate exhibited antimicrobial activity against both laboratory *E*. *coli* and human UTI *E*. *coli* isolates. In a gut simulator, salicylate reduced levels of *Enterobacteriaceae* and elevated *Bacteroidaceae* in a dose dependent manner.

## Introduction

Urinary tract infection (UTI) is the most prevalent infection observed in humans, affecting 150 million people annually worldwide [1]. While UTI can be caused by numerous species of bacteria, including *Klebsiella pneumoniae*, *Proteus mirabilis*, and *Staphylococcus aureus*, the opportunistic pathogen *Escherichia coli* accounts for the majority (>65%) of reported cases [1,2]. The human gut microbiota is a natural reservoir for uropathogenic *E*. *coli* (UPEC) which can colonize the perineal area and subsequently infect the urethra and urinary tract [3, 4]. Front line antibiotics (trimethoprim sulfamethoxazole, ciprofloxacin, and ampicillin) have historically been effective against *E*. *coli* and related organisms, but increasing rates of antibiotic resistance have led to reduced efficacy of these drugs, and concomitant increase in the rate of recurrence [1,5]. Further, antibiotics significantly impact the normal human microbiota, which in recent years has been shown to be an essential component of immune [6], metabolic [7], and even neural health [8]. The normal human gut microbiota also provide important colonization resistance against an array of pathogens [9, 10, 11].

There has been substantial research in expanding treatment options for UTIs beyond broad-spectrum antibiotics. This has led to the development of *E*. *coli*-specific vaccines or small molecules that target virulence factors like adhesion, which appears essential for the ability of *E*. *coli* to infect the urinary tract [12, 13]. Furthermore, there has been increasing interest in selectively eliminating UPEC from the gut reservoir; selective depletion of UPEC was accomplished using a FimH antagonist in mice colonized with UPEC without harming the gut microbiota [4]. These drugs however remain mostly in the preclinical or early clinical state and will take several years before potentially reaching patients.

As an alternative strategy, there has been an interest in exploring nutritional interventions which may decrease UTI recurrence rates and/or symptoms by reducing the natural reservoir of potentially UPEC. A popular remedy is the consumption (in the form of concentrate or juice) of the American cranberry, *Vaccinium macrocarpon*. While clinical trials exploring the beneficial effect of cranberry consumption on UTI recurrence and symptomologies show mixed results [14], several *in vitro* experiments have reported antimicrobial or antiadhesive properties of phenolic components of cranberry against *E*. *coli* [15, 16]. More recently, it was also found that organic acids at a high abundance in cranberries can reduce UTI in an animal model [17].

In this study, we sought to expand upon previous reports by broadly testing cranberry extracts and whole cranberry powder in a human gut microbiome-derived community in a gut simulator. We hypothesized that the reported, though inconsistent, reduction in UTI recurrence rates could be explained by cranberry-induced modulations on the gut microbiota community and a resulting reduction of *Enterobacteriaceae*, which could be uropathogenic. Here, cranberry components showed both promising prebiotic and modest antimicrobial activity, so we explored specific phenolic and non-phenolic cranberry components for anti-*E*. *coli* activity in monoculture. Candidates with attractive therapeutic activity profiles were tested in a human gut simulator seeded with a human gut microbiota. Cranberry-derived salicylate reduced *Enterobacteriaceae* in this model, while protecting core gut commensals.

## Results

### Cranberry extracts and whole cranberry powder modulate a human gut microbiota community

We hypothesized that cranberries, through prebiotic or antimicrobial activity, may alleviate UTI recurrence by reducing the host’s reservoir of UTI-causing *Enterobacteriaceae*. Thus, to determine the effect of cranberry consumption on the human gut microbiota composition, we treated stool communities each day for five days with 1 mg/mL phenolic-enriched or phenolic-deficient cranberry extracts or whole cranberry powder in a gut simulator. Because microbiota composition naturally varies between individuals [18], we seeded the gut simulator with a diverse human gut microbiota sample lacking *Enterobacteriaceae* (0.0% relative abundance *Enterobacteriaceae*) or with a sample with a high level of *Enterobacteriaceae* (15.7% relative abundance *Enterobacteriaceae*); *Enterobacteriaceae* level in the stool samples was determined by 16S rRNA gene sequencing. *Enterobacteriaceae* normally constitutes a small proportion of the healthy human gut microbiota at 0.1–1% relative abundance [18]. Both samples were collected from phenotypically healthy donors and represent the wide variation of the human gut microbiota.

**Fig 1:**
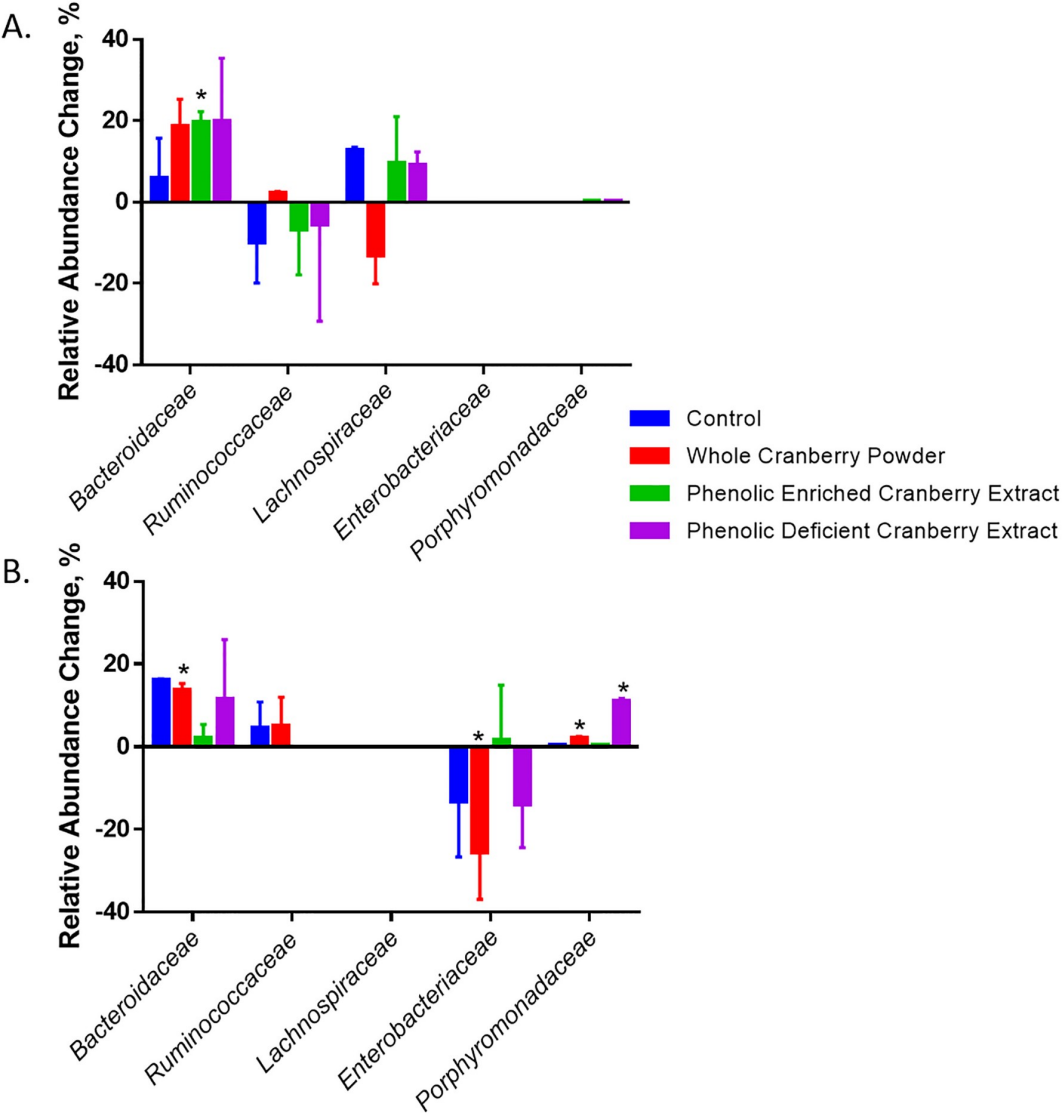
The average relative abundance changes of the most abundant families in a human gut simulator. A) A gut simulator seeded with a stool sample lacking *Enterobacteriaceae* B) or a stool sample high in *Enterobacteriaceae* and treated with whole cranberry powder (red), phenolic-enriched extract (green), or phenolic-deficient extract (purple) in duplicate. A gut simulator seeded with a human microbiota was treated each day for five days with 1 mg/mL of each extract or powder respectively. 16S rRNA gene sequencing was performed by an Ion Torrent PGM. The average relative abundance change was calculated from before treatment to after treatment with error bars representing standard deviation. Statistical significance (*) was calculated using the Student’s T-test for non-paired samples to compare the gut simulator community before treatment and after each respective treatment (p<0.05).

In the community derived from stool lacking *Enterobacteriaceae*, the phenolic-enriched extract significantly increased *Bacteroidaceae* (p = 0.028) while treatment with phenolic-deficient extract and whole cranberry powder increased the relative abundance of *Bacteroidaceae* approximately three-fold as compared to untreated controls (Fig 1A). Further, *Ruminococcaceae* and *Lachnospiraceae* relative abundance exhibited minor, insignificant fluctuations in all treatments (Fig 1A). This absence of fluctuation on important commensal taxa is a desirable outcome in regard to microbiome therapeutics. The phenolic-enriched and deficient extracts exhibited contrasting effects to whole cranberry powder. This suggests that various components of the cranberry work synergistically or antagonistically, making different cranberry components candidates for microbiota-specific targeted treatment.

We again observed treatment-dependent modulations when we tested the extracts and powder on a community derived from the high *Enterobacteriaceae* stool sample. Due to the difference in the composition of the stool inoculum, modulations in the dysbiotic stool sample were inconsistent from the non-dysbiotic stool sample, likely an example of donor-dependent effects of the treatment. In the gut simulator model inoculated with a high *Enterobacteriaceae* sample, the relative abundance of *Enterobacteriaceae* significantly decreases (p = 0.036) and stabilizes over time as the relative abundance of *Bacteroidaceae* increases (p = 0.011). Importantly, whole cranberry powder significantly reduced the relative abundance of *Enterobacteriaceae* (p = 0.0093) to a greater extent than control (Fig 1B). Further, whole cranberry powder significantly increased *Porphyromonadaceae* populations (p = 0.0019), and was able to maintain high levels of *Bacteroidaceae* over time, similar to the control. The phenolic-deficient extract significantly increased *Porphyromonadaceae* (p = 0.025) and stabilized *Enterobacteriaceae* to levels observed in the control (Fig 1B). Interestingly, the phenolic-enriched extract did not significantly alter the most abundant families of the dysbiotic community as compared to pre-treatment levels.

Based on these observations, the capability of cranberry components to alter the human gut microbiota composition by increasing the relative abundance of commensals and decreasing the relative abundance of *Enterobacteriaceae* was evident, but modulation was inconsistent and dependent on the baseline population of the donor sample. We reasoned that there are compounds present in cranberries that modulate the microbiota, but are at low and variable concentrations in our extracts, possibly below threshold for a consistent response. Thus, we aimed to determine which specific cranberry components have beneficial antimicrobial and prebiotic activity when tested at a defined concentration.

### Screen of cranberry components for antimicrobial activity against *Escherichia coli*

To explore potential antimicrobial activity of cranberry constituents against *Enterobacteriaceae*, a total of 44 cranberry fractions or purified compounds (Table 1) were tested for antimicrobial activity in a primary screen against *Escherichia coli* MG1655. The inhibition of growth was calculated for each compound, and we followed up with compounds that caused greater than 20% reduction of growth. Of the tested components, three purified compounds exhibited antimicrobial activity against *E*. *coli* MG1655 in the primary screen: salicylate, β-resorcylate, and t-Cinnamic acid. Generally, t-Cinnamic acid exhibited solubility issues, so it was dropped from further experimentation. Minimal inhibitory concentration (MIC) was determined for salicylate and β-resorcylate and found to be 1 mg/mL for both compounds against *E*. *coli* MG1655.

**Table 1:** The identity and preparation method of the fractions, or compounds tested for inhibition of *E*. *coli* MG1655 using the microbroth dilution Minimum Inhibitory Concentration assay.

Extract, Fraction, or Compound Identity	Preparation method
Quercetin-3-O-galactoside (heperoside)	Commercially purchased
Myricetin	Commercially purchased
Myricetin-3-O-galactoside	Commercially purchased
Myricitrin Dihydrate	Commercially purchased
Quercetin	Commercially purchased
quercitine-3-O-rhamnoside (Quercitrin)	Commercially purchased
Quercetin-3-Glucoside (Isoquercitrin)	Commercially purchased
Quinic acid	Commercially purchased
Benzoic Acid	Commercially purchased
p-Coumaric Acid	Commercially purchased
Chlorogenic Acid	Commercially purchased
Sinapic acid	Commercially purchased
Protocatechuic acid (3,4-Dihydroxybenzoic Acid)	Commercially purchased
Vanillic acid	Commercially purchased
Caffeic Acid	Commercially purchased
Ferulic Acid	Commercially purchased
t-Cinnamic Acid	Commercially purchased
Gallic Acid	Commercially purchased
2,4-Dihydroxybenzoic Acid	Commercially purchased
Salicylic acid (2/o-Hydroxybenzoic acid)	Commercially purchased
4-hydroxybenzoic Acid (p-hydroxybenzoic Acid)	Commercially purchased
3-hydroxybenzoic Acid (m-hydroxybenzoic Acid)	Commercially purchased
trans-2-hydroxycinnamic acid	Commercially purchased
Epicatechin	Commercially purchased
(+)-Catechin hydrate	Commercially purchased
procyanidin A2	Commercially purchased
procyanidin B2	Commercially purchased
Cranberry fraction 29	Phenolic enriched from cranberry concentrate
Cranberry fraction 30	Proanthocyanidin enriched from cranberry concentrate
Cranberry fraction 31	Crude oligosaccharide from cranberry juice powder
Cranberry fraction 32	Refine oligosaccharide from cranberry juice powder
Cranberry fraction 33	Phenolic enriched from cranberry juice powder
Cranberry fraction 34	Proanthocyanidin enriched from cranberry juice powder
Cranberry fraction 35	Crude oligosaccharide from cranberry concentrate
Cranberry fraction 36	Refine oligosaccharide from cranberry concentrate
Cranberry fraction 37	Phenolic enriched cranberry concentrate
Cranberry fraction 38	Proanthocyanidin enriched from cranberry concentrate
Cranberry fraction 39	Oligosaccharide from cranberry extract
Cranberry fraction 40	Phenolic enriched from cranberry extract
Cranberry fraction 41	Proanthocyanidin enriched from cranberry extract
Cranberry fraction 42	Oligosaccharide from cranberry concentrate 2
Cranberry fraction 43	Proanthocyanidin enriched from cranberry concentrate
Cranberry fraction 44	Oligosaccharide from cranberry pomace
Cranberry fraction 45	Proanthocyanidin enriched from cranberry pomace

### Anti-*Enterobacteriaceae* activity of salicylate and β-resorcylate in a human gut simulator

**Fig 2:**
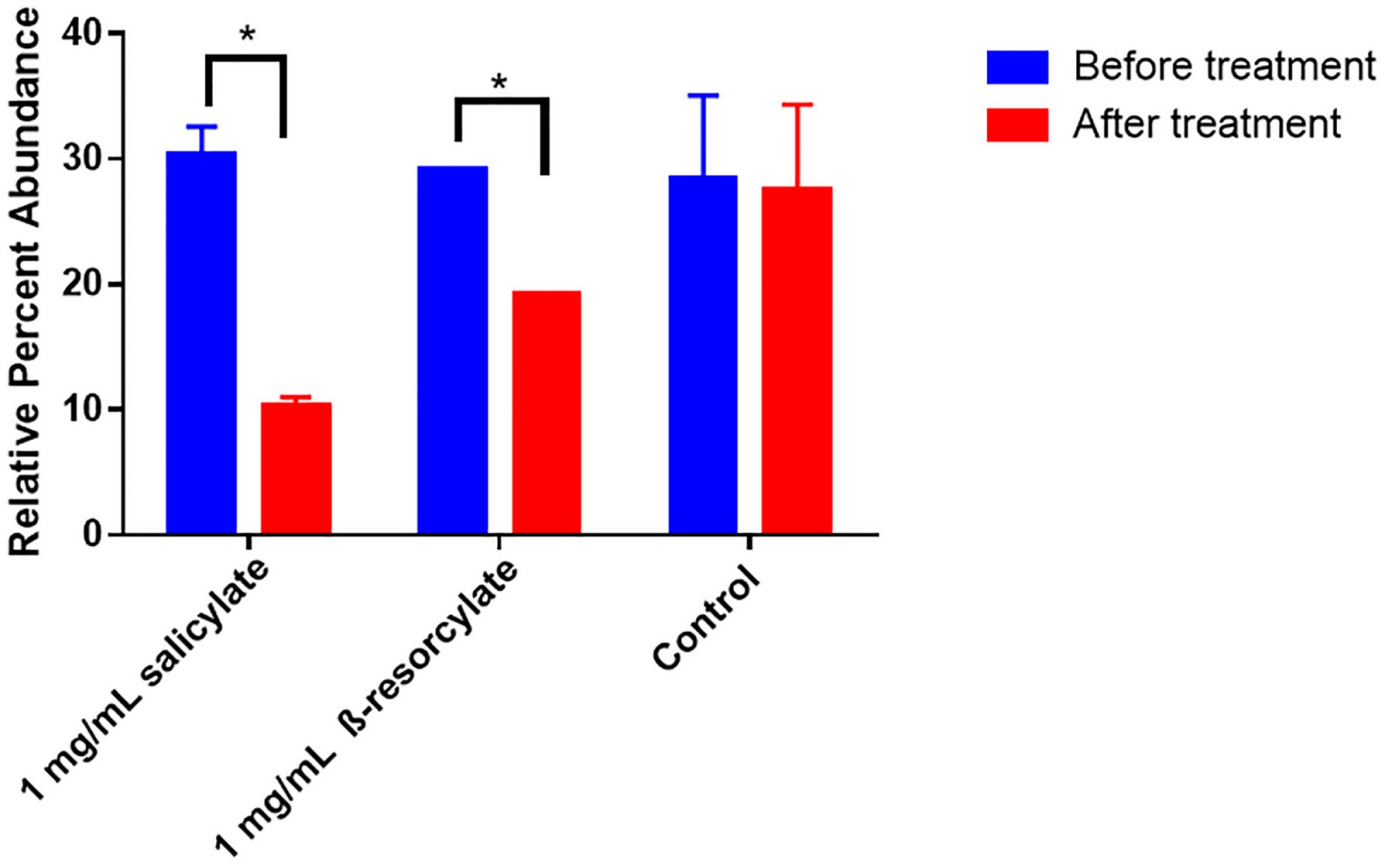
Relative abundance of *Enterobacteriaceae* in gut simulator before and after treatment with salicylate or β-resorcylate. A gut simulator seeded with a human gut microbiota was treated for five days with 1 mg/mL (1X MIC) of salicylate or β-resorcylate. All vessels had a 1% DMSO final concentration. 16S rRNA gene sequencing was performed by an Ion Torrent PGM. Salicylate and β-resorcylate significantly reduced the relative abundance of *Enterobacteriaceae*. β-resorcylate and control treatments were run in triplicate and the salicylate treatment was run in duplicate and averaged. Error bars represent standard deviation. Statistical significance (*) was calculated using the Student’s T-test for non-paired samples (p<0.05).

To investigate the effect of salicylate and β-resorcylate on *Enterobacteriaceae* in a human gut microbiota community, a gut simulator inoculated with human stool was treated each day for five days with 1X MIC salicylate or β-resorcylate dissolved in dimethyl sulfoxide (DMSO). DMSO can be used as a terminal electron acceptor and can cause *Enterobacteriaceae* blooms [19]. As such, to control for the bloom, all gut simulator vessels contained 1% DMSO. *Enterobacteriaceae* relative abundance were calculated before treatment and after five days of treatment. In previous experiments (Fig 1A and 1B), DMSO was not added and *Enterobacteriaceae* decreased over time; here, the addition of 1% DMSO stabilized the relative abundance of *Enterobacteriaceae* populations in control vessels. Five days of salicylate treatment reduced the *Enterobacteriaceae* relative abundance by 20.0% (p = 0.0074), compared to a 9.9% reduction by β-resorcylate (p = 0.0060) and a 0.8% reduction in control vessels (p = 0.89), as compared to pre-treatment levels (Fig 2). Although both salicylate and β-resorcylate significantly reduced the relative abundance of *Enterobacteriaceae*, the reduction by salicylate was two-fold greater than β-resorcylate, making it a more attractive candidate for further investigation as a modulator of the gut microbiota.

### Dose-dependent activity of salicylate in a human gut simulator

**Fig 3:**
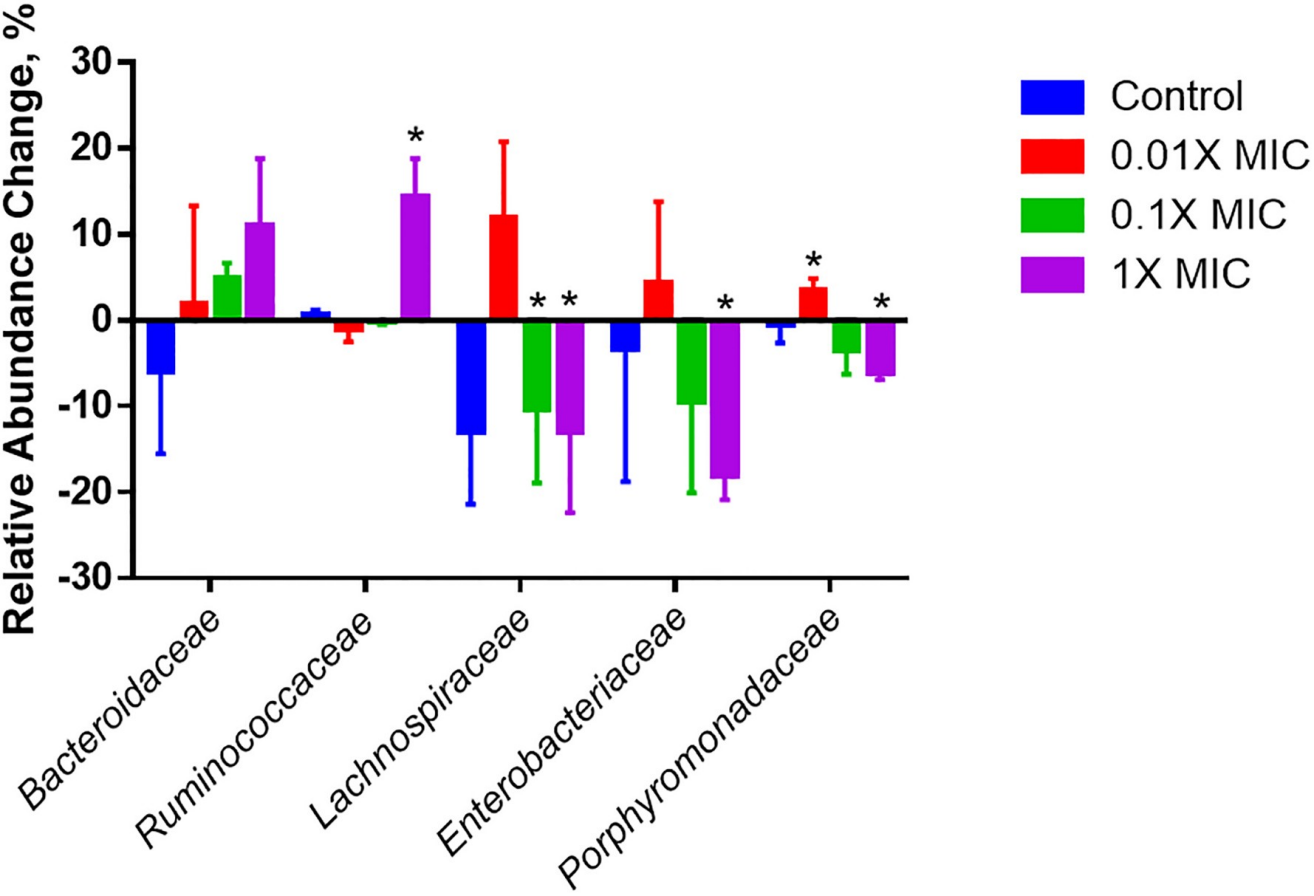
Average relative abundance change of five most abundant families in a gut simulator treated with salicylate. A gut simulator seeded with a human gut microbiota was treated with salicylate each day for five days at at 0.01 mg/mL (0.01X MIC, red), 0.1 mg/mL (0.1X MIC, green) and 1 mg/mL (1X MIC, purple). All vessels had a 1% final DMSO concentration. 16S rRNA sequencing was performed by an Ion Torrent PGM. Salicylate treatment increased *Bacteroidaceae* and decreased *Enterobacteriaceae* in a dose dependent manner. Experiment was performed in triplicate and the average percent abundance change from before treatment to after treatment was calculated. Error bars represent standard deviation. Statistical significance (*) was calculated using the Student’s T-test for non-paired samples to compare the gut simulator community before treatment and after each respective treatment (p<0.05).

To test the dose-dependence of salicylate as an anti-*Enterobacteriaceae* compound in a mixed community, a gut simulator was treated with 1X MIC (1 mg/mL), 0.1X MIC (0.1 mg/mL), or 0.01X MIC (0.01 mg/mL) salicylate. Following five days of treatment, salicylate exhibited a dose dependent response against *Enterobacteriaceae* (Fig 3), with only 1X MIC significantly decreasing the *Enterobacteriaceae* relative abundance over pre-treatment levels (18% reduction, 0 = 0.0095), compared to 0.1X MIC (9.4% reduction, p = 0.21), 0.01X MIC (4.3% reduction, p = 0.42) and the control (4% reduction, p = 0.34) (Fig 3). In order to validate these results from 16S rRNA gene sequencing analysis, samples from the gut simulator were collected and plated on selective MacConkey media to enumerate *Enterobacteriaceae* titers as CFU/mL (S1 Table). The final *Enterobacteriaceae* titers of 1X MIC treatment were about one log lower than in control vessels (p = 1.8E-07). Treatment with 0.1X MIC salicylate significantly decreased titers by one-half log (p = 2.1E-06), but 0.01X MIC treatment did not decrease titers significantly (p = 0.12); control titers did not change over time (p = 0.64) (S1 Table). Further, salicylate caused a dose-dependent relative abundance increase of *Bacteroidaceae*, and salicylate at 1X MIC significantly increased *Ruminococcaceae* (p = 0.0062) and *Lachnospiraceae* (p = 0.036) populations (Fig 3).

### Salicylate exhibits activity against clinically relevant *E*. *coli* UTI isolates

To determine whether salicylate has activity against clinically relevant *E*. *coli* isolates, the MICs for a panel of 12 isolates sourced from UTI patients were determined. The isolates were from patients treated with a broad array of antibiotics, yet all exhibited similar MICs to salicylate as *E*. *coli* MG1655, ranging from 0.5–2 mg/mL (Table 2).

**Table 2:** Minimum inhibitory concentration (MIC) of salicylate against *E*. *coli* MG1655 and clinical *E*. *coli* UTI isolates.

*Escherichia coli* strain	Salicylate minimum inhibitory concentration (mg/mL)
MG1655	1
W1	1
W9	1–2
W12	1
W19	1
W28	0.5–1
W29	1–2
W36	1
W42	1
W56	1–2
W67	0.5–1
W73	1
W81	1–2

## Discussion

Traditionally, it is thought that dietary ingestion of the American cranberry reduces UTI recurrence. Clinical trials on the efficacy of cranberry juice consumption in UTI prevention are conflicting, but suggest that women with recurrent UTI might receive a small benefit from cranberry juice consumption [14]. Furthermore, clinical trials on the efficacy of cranberry products on UTI recurrence have several limitations, including low patient adherence and low statistical power. If cranberry products do indeed provide a benefit to females with recurrent UTI, the reduction in UTI could be explained by a reduction in the natural gut reservoir of UPEC which in turn is influenced by the wider diversity of the gut microbiota. We reasoned that ingestion of certain cranberry components may modulate the microbiota through prebiotic and/or antimicrobial activity in a way that reduces the native reservoir of UTI-causing *Enterobacteriaceae*. Indeed, we observed that whole cranberry powder and phenolic-deficient and phenolic-enriched cranberry extracts altered a human gut microbiota community in a gut simulator. Modulations by these cranberry components tended to decrease *Enterobacteriaceae*, increase *Bacteroidaceae*, and stabilize *Ruminococcaceae* and *Lachnospiraceae* populations in our gut simulator model. This stabilization of the commensal families *Ruminococcaceae* and *Lachnospiraceae* is appealing when considering microbiota therapeutics. Whole cranberry powder promoted the greatest shift on *Enterobacteriaceae* levels in the simulated gut community, indicating that phenol and non-phenol compounds within cranberries may act synergistically to alter the gut microbiota.

Based on our observations of a shift from high abundance of *Enterobacteriaceae* to populations enriched with *Bacteroidaceae*, generally regarded as important members of a healthy microbiome [20], we screened a panel of cranberry constituents to identify specific compounds responsible. The panel included salicylate, which we found was a potent microbiota modulator that decreased *Enterobacteriaceae* and increased *Bacteroidaceae* and *Ruminococcaceae* levels in a dose-dependent manner. Salicylate has previously been reported to inhibit *E*. *coli* through disruption of translation, transcription, and translation [21]. Here, we expand on these reports by demonstrating the potential of salicylate to inhibit *Enterobacteriaceae* in a mixed, human gut microbiota-derived community while modulating the abundance of other symbiotic taxa.

Although the MIC of salicylate against *E*. *coli* MG1655 and a panel of *E*. *coli* UTI isolates is high relative to clinically relevant concentrations for antimicrobials, the ability to modulate the gut microbiota in microenvironments with long sustained exposure is worth considering. Salicylate decreased *Enterobacteriaceae* levels in a dysbiotic gut microbiota community in a gut simulator without negatively impacting core taxa. Thus, ingestion of cranberry-derived salicylate in combination with other components of cranberry may prophylactically lead to reduction in *E*. *coli* abundance, despite a high MIC against *E*. *coli*. Salicylate is bioavailable by dietary consumption and is present in a range of fruits and vegetables at varying concentrations [22]; however, it is known to be readily absorbed in the stomach [23] and achieving the MIC level of salicylate in the colon, even in a microenvironment of the colon, would indeed present a challenge. It should be noted that we did not determine if the benefits in a gut community resulting from salicylate treatment were due to salicylate antimicrobial activity against Enterobacteriaceae or due to prebiotic activity supporting commensals; it is possible that both activities contribute to the shifts observed in the community and likely explain the shifts observed with cranberry extract containing lower concentrations of salicylate.

While a decrease in *Enterobacteriaceae* may be beneficial in reducing the native reservoir of UTI-causing bacteria, decreasing *Enterobacteriaceae* may otherwise be relevant. For example, high fecal *Enterobacteriaceae* levels are reported in patients with inflammatory bowel diseases [19]. Gram-negative members of the *Enterobacteriaceae* family have lipopolysaccharide in their outer membrane, which signals a pro-inflammatory response through the Toll-like receptor 4-MD2-CD14-LBP transduction pathway [24, 25]. Intestinal inflammatory conditions can support the growth of *Enterobacteriaceae* pathobionts, which exploit the availability of host-derived factors (e.g. nitrates, trimethylamine N-oxide, DMSO) for anaerobic respiration and successful competition over strictly anaerobic members of the gut microbiota [26]. Dysbiosis characterized by an *Enterobacteriaceae* bloom can further promote the immune imbalance and exacerbate the inflammatory status of the gut epithelium [27]. Thus, ingesting cranberry extract or salicylate, with additional measures for targeted delivery of high concentrations to the colon, may represent a strategy to reduce *Enterobacteriaceae*-induced inflammation. Furthermore, a consistent increase in *Bacteroidaceae* populations was a notable outcome of salicylate and whole cranberry powder treatment on human gut microbiota populations. We have previously shown that *Bacteroidaceae* are negatively correlated with depression, which may be due to their ability to produce the neurotransmitter gamma-Aminobutyric acid (GABA) [28].

Taken together, our studies suggest that ingestion of components from the American cranberry modulate the microbiota in a manner that may be beneficial by enriching *Bacteroidaceae* and reducing *Enterobacteriaceae* levels in the gut microbiota. While several compounds likely act synergistically in the cranberry to drive *in vitro* microbiota compositional changes, we identify cranberry-derived salicylate as a beneficial modulator of the human gut microbiota.

## Materials and methods

### Ethics statement

The Northeastern University Institutional Review board (Cultivating Unculturable Bacteria from the Human Microbiome: Feces, number 08-11-16) approved the collection of feces from human subjects. Written consent was obtained from donors.

### Stool sample collection

Stools were collected at Northeastern University following an Institutional Review Board-approved protocol (number 08-11-16). Briefly, one fresh stool sample was collected from each healthy adult donor (n = 26) using stool collection vessels (Medline Industries). Donors were not currently taking antibiotics and had not taken antibiotics for at least two weeks at the time of collection. Using the attached scoop, a sample of the stool was immediately placed in 9 mL pre-reduced PBS (24 hours in anaerobic chamber) to total approximately 10 mL of slurry in 50 mL collection tubes (Fisher Scientific). The stool slurry was homogenized in a Coy Anaerobic Vinyl chamber (Coy Laboratory Products, Inc.) in 5% hydrogen, 10% CO_2_, 85% nitrogen at 37°C. Samples were aliquoted (1 mL) in 20% glycerol and stored at -80°C.

### Culture media recipes

GIFU Anaerobic Medium broth (GIFU, HIMedia) was prepared according to the manufacturer’s instructions and then diluted and buffered as follows to yield greater population diversity [29]. Deionized water was sterilized by autoclaving for 60 minutes at 121°C/20 PSI. Filter sterilized 1.5 M solution of 3-(N-morpholino)propanesulfonic acid (MOPS) buffer was adjusted to pH 7.0 using 10 M NaOH. The individual components were combined aseptically to obtain a 1:10 dilution of the GIFU broth in water, buffered with a 0.5 M MOPS working solution, and reduced in an anaerobic chamber for 48 hours on a stir plate at 200 rpm prior to use.

### Preparation of cranberry extracts, fractions, and pure compounds

Whole cranberry powder, the phenolic-enriched and phenolic-deficient extracts, and cranberry fractions were prepared from a mixture of cranberry cultivars, primarily the Stevens variety. Whole cranberry powder was prepared by grinding cranberries frozen in liquid nitrogen into a powder.

To prepare the phenolic-enriched extract, whole cranberry powder was solubilized in deionized water; to prepare phenolic-enriched fractions from concentrate or pomace, cranberry concentrate or ground pomace were solubilized in deionized water; juice was not further solubilized. Solubilized cranberry material or juice was filtered with a 0.7um GMF filter (Thermo Titan 3 30mm Filter (0.7μm GMF Membrane)) and injected into the Flash Chromatography System (Agilent Flash Purification System (971-FP) together with the Biotage SNAP Cartridge (KP-C18-HS 120g)). The following gradient was used for elution of oligosaccharides and phenolic compounds with water (A) and 0.1% acetic acid methanol (B) as mobile phases: 0–10 min 100% A; 10–20 min 85% A; 20–30 min 100% B. The 15% methanol fraction and the 100% methanol fraction which contained phenolic compounds were collected respectively in glass bottles. The 100% methanol fraction was further applied onto the LH-20 column (Lipophilic Sephadex LH20 Media, Sigma LH20–1000). The liquid sample was allowed to flow into the bed, then 10–20ml of deionized water as starting mobile phase was added to the bed and flowed through making sure the sample solution was completely washed into the bed. Ethanol (25%) was used to elute and collect the phenolic-enriched fraction. The composition of the phenolic-enriched extract is reported in S2 Table. To prepare the phenolic-deficient extract, the 15% methanol was further applied onto the LH-20 column. Deionized water was used as the starting mobile phase and elution solvent. The proanthocyanidin fraction was collected as previously described as a 70% acetone fraction [30].

The 44 fractions or pure compounds were commercially purchased based on cranberry composition or prepared from cranberry juice powder, cranberry concentrate, and cranberry pomace (Table 1).

### Operation of the gut simulator

A modified simulator of the human intestinal microbial ecosystem (SHIME) [31] was used to culture human gut communities in dGIFU broth. The modified simulator (Lewis Gut Simulator, LEGS) consisted of one reactor to represent just the colon community, compared to the SHIME model’s 5 reactor system representing the duodenum to the ascending colon. LEGS consisted of a bottle of sterile broth which was pulled through a sterile metal straw and sterile silicone tubing (0.062” ID x 0.188” OD). This tubing was connected via a straight connector to sterile silicone tubing (0.093” ID x 0.157” OD) which was branched using T-connectors (3/16” ID) into four discrete paths connected to peristaltic tubing (size 0.89 mm) on a pump (Ismatec ISM404 Pump). The peristaltic tubing output was connected to additional sterile silicone tubing (0.062” ID x 0.188” OD) which flowed into vessel chambers. Peristaltic tubing connections were made with reduction couplers. The chambers were custom made from 400 mL beakers (Yankee Glassblowers) and fitted with a nylon 3D printed lid with ports for medium input at the top and output at the side at 150 mL. The LEGS system was sterilized by autoclaving and assembled in a vinyl anaerobic chamber. Media were pre-reduced for 48 hours before being loaded in the system to fill each entire vessel volume (150 mL). A stool sample diluted 10^−6^ in deoxygenated medium was inoculated into the vessel chambers and then media was pumped at 0.101 mL/minute so that 145.44 mL was replaced every 24 hours. The microbial population established for 48 hours prior to treatment interventions. Treatments were administered every 24 hours for five days by dosing directly into each chamber vessel. Phenolic-enriched and phenolic-deficient extracts and whole cranberry powder were dosed at 1 mg/mL; β-resorcylate was dosed at 1X MIC (1 mg/mL) and salicylate was dosed at 1X MIC (1 mg/mL), 0.1X MIC, or 0.01X MIC. Sampling was conducted before and after treatment interventions. The average relative abundance change was calculated by averaging the percent relative abundance change of a given taxa in each replicate from before treatment intervention to after treatment intervention. Samples were stored at -80°C for sequencing and plated on pre-reduced MacConkey agar using sterile glass beads to calculate the CFU/mL of Enterobacteriaceae. Plates were incubated anaerobically for 48 hours. All experiments were performed at 37°C in an environment containing a gas mixture of 85% Nitrogen, 10% CO_2_, and 5% Hydrogen. The phenolic-enriched or deficient extracts and whole cranberry powder were tested on stool from two different donors in duplicate (low Enterobacteriaceae sample) or triplicate (high Enterobacteriaceae sample). All β-resorcylate and salicylate treatments were performed on stool from a single donor in duplicate (β-resorcylate) or triplicate (salicylate, salicylate dose-dependence).

### 16S rRNA gene sequencing and processing

Sequencing was performed by MR DNA (www.mrdnalab.com, Shallowater, TX, USA) on an Ion Torrent PGM. The V4 variable region was amplified using PCR primers 515/806 (515F: GTGCCAGCMGCCGCGGTAA, 806R: GGACTACVSGGGTATCTAAT) in a single-step 30 cycle PCR with the HotStarTaq Plus Master Mix Kit (Qiagen, USA). The following conditions were used: 94°C for 3 minutes and 30 cycles of 94°C for 30 seconds, 53°C for 40 seconds and 72°C for 1 minute, followed by a final elongation step at 72°C for 5 minutes. A proprietary analysis pipeline (MR DNA, Shallowater, TX, USA) was used to process the data. Barcodes and primers were removed and then sequences with less than 150 bp were removed. Sequences with ambiguous base calls or with homopolymer runs greater than 6bp were removed. The sequences were denoised. Operational taxonomic units (OTUs) were generated and chimeras were removed. OTUs were defined by 97% similarity and were taxonomically classified against a database derived from RDPII (http://rdp.cme.msu.edu) and NCBI (www.ncbi.nlm.nih.gov).

### Minimum Inhibitory Concentration (MIC) assay

The microbroth dilution Minimum Inhibitory Concentration (MIC) method was used to quantitatively measure the *in vitro* antibacterial activity of 44 cranberry fractions or pure compounds (Table 1) against *E*. *coli* MG1655.

To test the activity of cranberry components, a single colony of *E*. *coli* MG1655 was grown overnight in LB broth (Fisher BioReagents, USA). The overnight culture was diluted 1:100 and grown aerobically to OD600 0.5 at 37°C. Cranberry compounds were solubilized in 100% DMSO (10 mg/mL). Compound solutions were diluted to 200 μg/mL in LB broth. The OD600 0.5 culture was diluted 1:100 and added in 1:1 ratio with the compound solution. Cultures were incubated aerobically at 37°C overnight. OD600 was read on a BioTek Synergy H1 microplate reader and percent inhibition was calculated by comparing the OD600 of the treated cultures to the OD600 values of the untreated culture.

The MIC of soluble cranberry compounds that inhibited growth by 20% or more was calculated against *E*. *coli* MG1655. The culture (OD600 0.5) was diluted 1:500 into a 1:2 dilution series in LB broth of salicylate or β-resorcylate (solubilized in 100% DMSO, 100 mg/mL) and incubated aerobically or anaerobically overnight at 37°C. The MIC was calculated as the lowest drug concentration with total inhibition of visual growth.

### Strain list

*E*. *coli* isolates sourced from patients treated with antibiotics for UTIs were obtained from Dr. Anne Stapleton at the University of Washington. Twelve isolates were randomly chosen for MIC testing (Table 3).

**Table 3:** *E*. *coli* isolates sourced from patients with UTI before treatment with antibiotics.

Genus and species	Strain name ID	Date isolated	Patient disease	Patient antibiotic course
*Escherichia coli*	W1	02/06/2003	UTI (Cystitis)	Trimethoprim/Sulfamethoxazole
*Escherichia coli*	W9	12/2/2003	UTI (Cystitis)	Nitrofurantoin
*Escherichia coli*	W12	4/13/2004	UTI (Cystitis)	Trimethoprim/Sulfamethoxazole
*Escherichia coli*	W19	2/24/2005	UTI (Cystitis)	Trimethoprim/Sulfamethoxazole
*Escherichia coli*	W28	11/17/2005	UTI (Cystitis)	Trimethoprim/Sulfamethoxazole
*Escherichia coli*	W29	1/3/2006	UTI (Cystitis)	Nitrofurantoin
*Escherichia coli*	W36	5/1/2006	UTI (Cystitis)	Trimethoprim/Sulfamethoxazole
*Escherichia coli*	W42	1/2/2003	UTI (Cystitis)	Nitrofurantoin
*Escherichia coli*	W56	10/19/2007	UTI (Cystitis)	Cefpodoxime
*Escherichia coli*	W67	5/23/2003	Recurrent UTI	Ciprofloxacin
*Escherichia coli*	W73	8/13/2003	Recurrent UTI	Trimethoprim/Sulfamethoxazole
*Escherichia coli*	W81	8/13/2004	Recurrent UTI	Ciprofloxacin

### Statistical analysis

Statistical analysis was performed with the Student’s T-test with a two-tailed distribution for non-paired samples. In the gut simulator, the community before treatment was compared to the community after each respective treatment.

## Supporting information

S1 Table *Enterobacteriaceae* titers before and after salicylate treatment of a human gut microbiome community. A human gut microbiome community in a gut simulator was treated with 0.01X, 0.1X, and 1X MIC salicylate. *Enterobacteriaceae* titers were determined by plating for CFU on MacConkey agar. Salicylate treatment at 1X MIC and 0.1X MIC significantly reduced the CFU/mL compared to the control, in a dose-dependent manner.
(DOCX)

S2 Table Analytical composition of the phenolic-enriched cranberry extract. Methods used were previously described [30].
(DOCX)
