# Supplementary material for: Cranberry extracts promote growth of Bacteroidaceae and decrease abundance of Enterobacteriaceae in a human gut simulator model
Source: PLoS One. 2019 Nov 12;14(11):e0224836. doi: 10.1371/journal.pone.0224836 (PMC6850528; doi:10.1371/journal.pone.0224836)
Supplement: S1 Table — A human gut microbiome community in a gut simulator was treated with 0.01X, 0.1X, and 1X MIC salicylate. Enterobacteriaceae titers were determined by plating for CFU on MacConkey agar. Salicylate treatment at 1X MIC and 0.1X MIC significantly reduced the CFU/mL compared to the control, in a dose-dependent manner. (DOCX) [file pone.0224836.s001.docx]

**S1 Table. *Enterobacteriaceae* titers before and after salicylate treatment of a human gut microbiome community**. A human gut microbiome community in a gut simulator was treated with 0.01X, 0.1X, and 1X MIC salicylate. *Enterobacteriaceae* titers were determined by plating for CFU on MacConkey agar. Salicylate treatment at 1X MIC and 0.1X MIC significantly reduced the CFU/mL compared to the control, in a dose-dependent manner.

| Treatment | *Enterobacteriaceae* CFU/mL before treatment | *Enterobacteriaceae* CFU/mL after treatment |
| --- | --- | --- |
| 1X MIC salicylate | 1.03x10^8^ | 1.62 x10^7^ |
| 0.1X MIC salicylate | 1.07x10^8^ | 4.55x10^7^ |
| 0.01X MIC salicylate | 1.13x10^8^ | 9.23x10^7^ |
| Control | 1.10x10^8^ | 1.17x10^8^ |
