# Supplementary material for: Cranberry extracts promote growth of Bacteroidaceae and decrease abundance of Enterobacteriaceae in a human gut simulator model
Source: PLoS One. 2019 Nov 12;14(11):e0224836. doi: 10.1371/journal.pone.0224836 (PMC6850528; doi:10.1371/journal.pone.0224836)
Supplement: S2 Table — Methods used were previously described [30]. (DOCX) [file pone.0224836.s002.docx]

**S2 Table. Analytical composition of the phenolic-enriched cranberry extract.** Methods used were previously described [31]. ND indicates not detected.

|  | **Phenolics enriched fraction (range)** | **Phenolics enriched fraction (average)** |
| --- | --- | --- |
|  | **As is %** | **As is %** |
| Solids | **93.0-96.9** | **95%** |
| **Total Anthocyanins** | **1.63-3.56** | **2.35** |
| Cyanidin-3-Arabinoside | 0.34-0.61 | 0.44 |
| Cyanidin-3-Galactoside | 0.37-0.89 | 0.62 |
| Cyanidin-3-Glucoside | 0.02-0.03 | 0.02 |
| Peonidin-3-Arabinoside | 0.27-0.62 | 0.40 |
| Peonidin-3-Galactoside | 0.13-0.51 | 0.79 |
| Peonidin-3-Glucoside | 0.06-0.12 | 0.08 |
|  |  |  |
| **Total Organic Acids** | **0.6-3.9** | **2.28** |
| Citric Acid | 0.43-3.39 | 1.78 |
| Galacturonic Acid | ND | ND |
| Malic Acid | 0.32-1.91 | 1.05 |
| Quinic Acid | 0.28-2.17 | 1.22 |
|  |  |  |
| **Total Flavan-3-ols** | **0.72-1.1** | **0.88** |
| Catechin | 0.15-0.21 | 0.18 |
| Epicatechin | 0.57-0.89 | 0.70 |
|  |  |  |
| **Total Flanovols** | **5.16-11.3** | **8.28** |
| Myricetin | 0.13-0.42 | 0.25 |
| Myricetin-3-galactoside | 0.87-1.77 | 1.35 |
| Myricitrin | 0.12-0.66 | 0.43 |
| Quercetin | 0.40-0.86 | 0.60 |
| Quercitrin | 0.68-1.11 | 0.82 |
| Hyperoside | 2.05-4.56 | 3.35 |
| Other flavonols | 0.91-1.92 | 1.49 |
|  |  |  |
| **Total phenolic acids** | **9.6-16.4** | **11.84** |
| Chlorogenic Acid | 0.74-1.26 | 0.95 |
| Gallic Acid | 0.01-0.02 | 0.01 |
| 3,4-Dihydroxybenzoic acid | 0.05-0.18 | 0.11 |
| Benzoic Acid | 2.41-5.04 | 3.47 |
| Caffeic Acid | 0.07-0.19 | 0.10 |
| Vanillic Acid | 0.07-0.21 | 0.13 |
| p-Coumaric Acid | 0.46-1.05 | 0.68 |
| t-Cinnamic Acid | 0.07-0.12 | 0.09 |
| Ellagic acid | ND | ND |
| Ferulic acid | 0.04-0.08 | 0.06 |
| Salicylic acid | ND | ND |
| Other phenolic acids | 5.68-8.22 | 6.24 |
|  |  |  |
| Proanthocyanidins |  |  |
| BL-DMAC | 13.9-18.3 | 16.26 |
| OSC-DMAC | 52.3-56.3 | 54.50 |
|  |  |  |
| **Total Sugars** | **0-7.34** | **1.62** |
| Dextrose | 0-0 | 0.00 |
| Fructose | 0-7.34 | 1.62 |
|  |  |  |
| **Total Phenolics by Folin** | **38.5-56.8** | **47.31** |
